# Variation in rates of self-harm hospital admission and re-admission by ethnicity in London: a population cohort study

**DOI:** 10.1007/s00127-021-02087-9

**Published:** 2021-04-20

**Authors:** C. Polling, I Bakolis, M. Hotopf, S. L. Hatch

**Affiliations:** 1 Department of Psychological Medicine, Institute of Psychiatry, Psychology and Neuroscience, King’s College London, 16 De Crespigny Park, PO Box 84, London SE5 8AF, UK; 2 South London and Maudsley NHS Foundation Trust, London, UK; 3 Department of Biostatistics and Health Informatics, Institute of Psychiatry, Psychology and Neuroscience, King’s College London, London, UK

**Keywords:** Self-harm, Suicide, Ethnicity, Socio-economic status, Epidemiology

## Abstract

**Purpose:**

To compare sex-specific rates of hospital admission and repeat admission following self-harm between ethnic groups in London and test whether differences persist after adjustment for socio-economic deprivation.

**Methods:**

A population-based cohort of all individuals aged over 11 admitted to a general hospital for physical health treatment following self-harm between 2008 and 2018, using administrative Hospital Episode Statistics for all people living in Greater London.

**Results:**

There were 59,510 individuals admitted to the hospital following self-harm in the 10 year study period, ethnicity data were available for 94% of individuals. The highest rates of self-harm admission and readmission were found in the White Irish group. Rates of admission and readmission were lower in Black and Asian people compared to White people for both sexes at all ages and in all more specific Black and Asian ethnic groups compared to White British. These differences increased with adjustment for socio-economic deprivation. People of Mixed ethnicity had higher rates of readmission. Rates were highest in the 25–49 age group for Black and Mixed ethnicity men, but in under-25 s for all other groups. There were substantial differences in rates within the broader ethnic categories, especially for the Black and White groups.

**Conclusion:**

In contrast to earlier UK studies, self-harm rates were not higher in Black or South Asian women, with lower self-harm admission rates seen in almost all ethnic minority groups. Differences in rates by ethnicity were not explained by socio-economic deprivation. Aggregating ethnicity into broad categories masks important differences in self-harm rates between groups.

## Introduction

Self-harm, through both self-injury and overdose [1], has affected over 6% of the population in England at some point in their lifetime [2] and results in over 100,000 hospital admissions [3] each year in England. Work from the USA has demonstrated differences in rates of self-harm and suicide in different racial and ethnic groups [4]. Chu et al. [4] identified that cultural influences may shape whether and what types of stressors prompt self-harm and suicidal behaviour and how these behaviours are expressed. Other authors have highlighted the importance of structural disadvantage and discrimination experienced by minority groups in driving health disparities, including for mental health outcomes [5].

In the UK, general population surveys, including the most recent Adult Psychiatric Morbidity Survey, have typically been underpowered to detect differences in self-harm between ethnic groups [2, 6]. Research comparing rates of self-harm has instead overwhelmingly relied on the larger datasets generated by service use data. A systematic review in 2007 found that, compared to White women, South Asian women had higher rates of self-harm, while South Asian men had lower rates compared to White men. For repetition of self-harm, the South Asian and Caribbean populations of both sexes had lower rates than the White population [7]. More recent studies in a 2015 systematic review [8] have suggested that self-harm rates may no longer be raised in South Asian women but two studies, one in Manchester, Derby and Oxford [9] and the other in London [10] have suggested they may be raised in young Black women. Conversely, recent area-level studies in South East London [11] and Manchester [12] have suggested that rates of self-harm may be lower in areas with higher non-White British populations.

Overall the picture of how rates of self-harm vary by ethnicity remains unclear. Studies have not had sufficient numbers to examine the role of ethnicity beyond very broad and heterogenous categories that may mask important differences between groups. Most have also not considered the potentially significant confounding role of low socio-economic position, which is associated with self-harm at both individual and area level [13] and is more common in many ethnic minority populations.

### Aims

This study examined general hospital admissions following self-harm by people over the age of 11 resident in Greater London from 2008 to 2018. It aimed to test (1) whether rates of hospital admission for self-harm differ by ethnicity; (2) whether these differences are explained by the deprivation of the areas in which people live and (3) whether rates of repeat admission for self-harm within a year of the first admission differ by ethnicity. We hypothesised (1) that rates of self-harm would differ between ethnic groups; (2) that any differences would be partially, but not wholly, explained by socio-economic position; and (3) that rates of repetition would be higher in the White British group.

## Methods

### Study area

The study area is the Government Office for the London region, an area with a population of 8.1 million at the 2011 census. It is diverse in terms of ethnicity, with more than half the population not identifying their ethnicity as White British. The largest ethnic minority groups are White Other (13.2%), Indian (6.8%) and Black African (6.6%). There is also a great deal of variety in socio-economic position. This study uses the Index of Multiple Deprivation (IMD), a composite measure which combines domains relating to income, employment, education, housing, health, crime and living environment to summarise the multiple dimensions of deprivation within small-areas in England [14]. It is not an absolute measure but describes areas’ deprivation relative to each other, hence quantiles rather than absolute scores are used to compare areas [15]. Within London, areas rank between the 2nd and 100th centiles relative to England as a whole. Overall, over half London’s population live in areas that rank in the two most deprived quintiles nationally. Supplementary Table 1 shows the ethnic make-up of London’s population by area deprivation and demonstrates that people of Black Caribbean, Black African, Black Other and Bangladeshi ethnicity are particularly likely to live in the most deprived areas.

### Data sources

#### Main analysis

The data source for the main analyses was Hospital Episode Statistics (HES), routine administrative data collected by all National Health Service (NHS) hospitals in the UK [16]. HES data was provided by NHS Digital in anonymised format. We used the Admitted Patient Care (APC) dataset, which includes all inpatient admissions to general (physical health care) hospitals but does not include Emergency Department attendances that do not result in admission. Each episode is given diagnostic codes according to the ICD-10 [16], including “external cause” codes relating to the cause of an injury or illness. Self-harm was defined as an emergency (unplanned) admission to a general hospital with an ICD-10 diagnostic code in the range X60-X84 (Intentional self-harm) [17], to match the definition used by Public Health England for its Public Health Outcomes Framework [18]. This represents admission for treatment of the physical health consequences of self-harm.

The full list of ICD-10 codes included is given in Supplementary Table 2. Codes starting X60-X69 were grouped together as Self-poisoning; codes X72-X79 as Self-injury, including use of sharp and blunt objects and burning; codes X70-X71 and X80-X83 were grouped as Other, this included hanging, drowning, jumping from heights, and finally X84 codes were grouped as Unspecified. All episodes between 1/4/2008 and 31/3/2018 for individuals aged 11 or over who gave a home address within the Government Office for London Region at the time of admission were included, regardless of the location of the hospital they were admitted to. Each individual’s first admission during the study period was included in the analysis.

Ethnicity, age, sex and Lower Super Output Area of residence (LSOA) were taken from HES data. Ethnicity was provided in the 16 categories used in the 2001 census, hence the categories in the 2011 census denominator data were merged where necessary to reflect this. Deprivation was measured using the IMD 2015 of individuals’ LSOA of residence and divided into deciles based on their national rank. IMD scores for each LSOA were downloaded from the Greater London Authority (GLA) Datastore LSOA Atlas [19].

#### Sensitivity analysis

A sensitivity analysis was conducted using a previously created dataset of Emergency Department (ED) attendances following self-harm. Data were available for presentations between 1/4/2009 and 31/3/2016 to four South East London general hospitals’ EDs by people with a home address in one of the four London boroughs these hospitals serve: Lambeth, Southwark, Lewisham and Croydon. This data differs from HES APC data because it includes all ED presentations, regardless of whether or not they led to a hospital admission, however it relates to a more limited geographical area and time period. The dataset was created using electronic patient records linked to Hospital Episode Statistics, the full methodology has been published elsewhere [20].

#### Population denominator

Population age distribution varies by ethnicity in London. Rates of self-harm also vary substantially by age, hence a valid comparison of rates by ethnicity requires age stratification or standardisation. Data for the denominator population, broken down by ethnicity, age and sex was taken from the 2011 census. This data is available at LSOA level separated by ethnicity, age in four bands and sex [21]. This was used for the creation of denominator populations by deprivation quantile. Census data was accessed via the Nomis website [22].

### Statistical analyses

#### Rates of admission by ethnicity

All cases with complete data for age, sex and ethnicity were included. Rates of first admission for self-harm were calculated for the five broad census ethnicity categories, disaggregated by sex and age. Rates were then directly standardised for IMD decile using the total London population as the standard. Rate ratios were calculated comparing other ethnic groups to the White group and difference by ethnicity tested using likelihood ratio tests. Rates were then calculated for each of the 16 census 2011 ethnic groups, disaggregated by sex, directly standardised first for age and then for IMD decile. Rate ratios were calculated using White British as the reference group.

Rates of repetition were calculated for the 12 months after an individuals’ first presentation, excluding those people who did not have 12 full months of follow-up, hence only those who were admitted for the first time between 1/4/2008 and 31/3/2017 were included in this analysis. Rate ratios were calculated for each ethnic group compared to White British using log-binomial regression, first in univariate analyses, then adjusted for age, sex, type of self-harm and area deprivation in national deciles.

#### Sensitivity analysis

Data on ED attendances in South East London were used to calculate age-standardised rates of the first ED presentation. These were compared to rates of admission in the same geographical area and time period. Due to the smaller numbers in this dataset, ethnicity was grouped into 6 categories: White British, White non-British, Mixed, Asian, Black and Other.

Analyses were carried out and figures produced in R version 3.6.0.

## Results

Over the 10 year period studied, there were 85,139 admissions to the hospital for self-harm by 59,510 individuals living within the study area. Information on ethnicity was available for 81,579 (95.8%) of admissions and 56,117 (94.3%) individuals. The overall rate of the first admission was 9.8/10,000 (9.7–9.9) for females and 6.1/10,000 (6.0–6.2) for males.

### Rates of admission by ethnicity

**Fig. 1:**
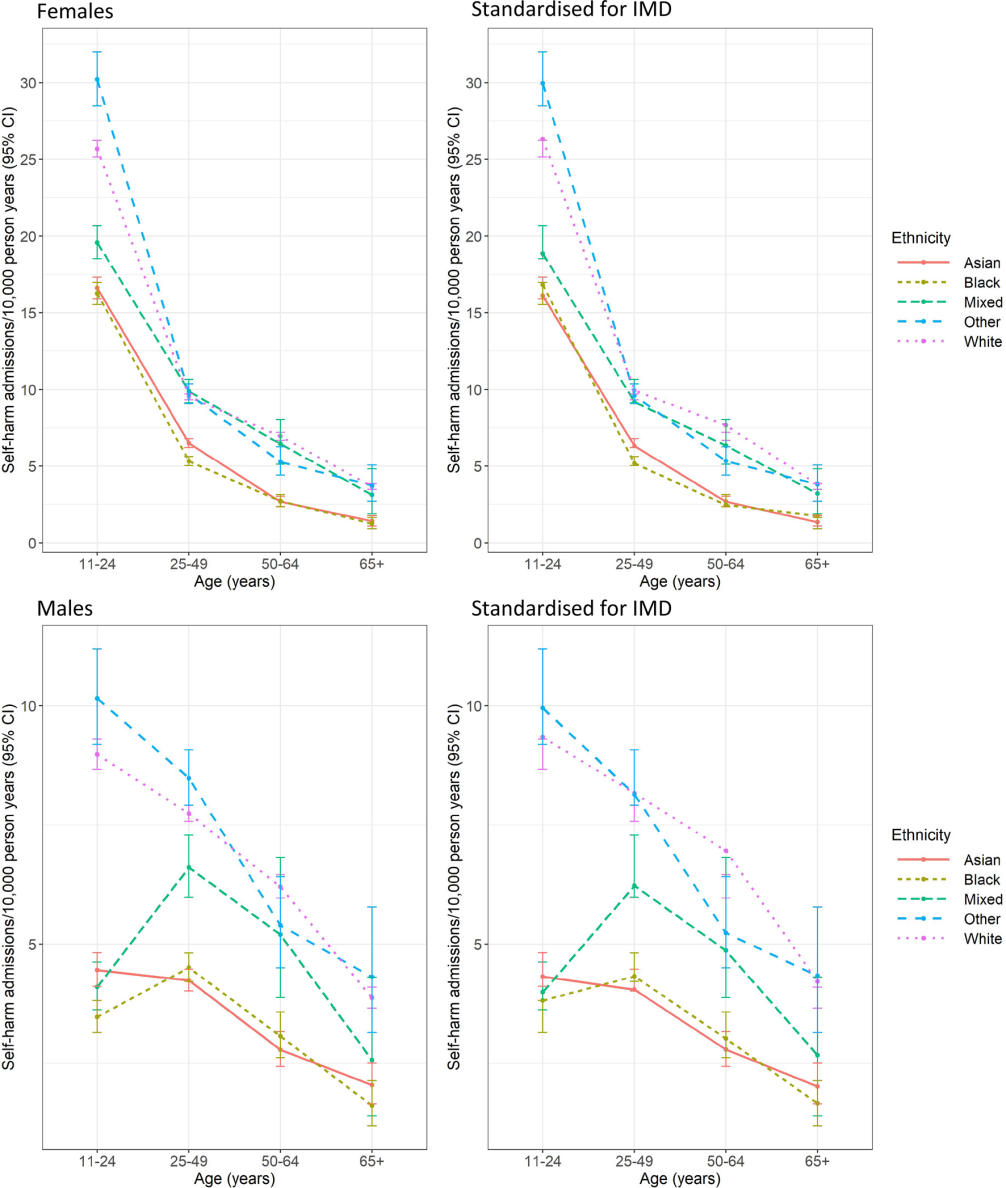
Rates of admission for self-harm by ethnicity and age, London 2008–2018

Figure 1 and Supplementary Table 3 show the distribution of first admissions by sex, age and ethnicity. Rates of admission for self-harm in both the Black and Asian groups were below those of the White group at all ages in both sexes both before and after standardisation for deprivation. The Mixed ethnicity group had rates closer to the White group in the age groups over 25, although their rates decreased, increasing the difference from the White group, with standardisation for deprivation. In women of all ethnicities, rates of self-harm were highest in the 11–24 age group and declined with age. This was also the case for White, Asian and Other men, however, in the Black and Mixed ethnicity groups male rates were highest in the 25–49 age group.

**Fig. 2:**
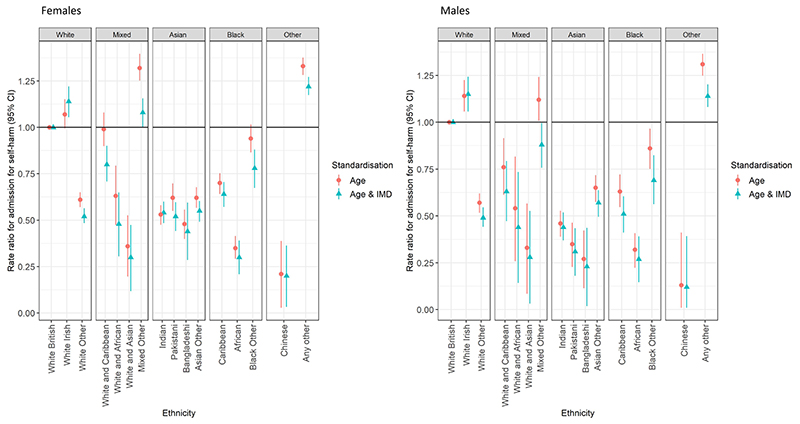
Rate ratios for admission following self-harm by ethnicity, London 2008–2018

Figure 2 and Table 1 show age and IMD-standardised rate ratios for self-harm admission by more detailed ethnic group, demonstrating considerable variation within the broader ethnic groups used in Fig. 1. For example, within the White group, the White Other population had much lower rates than the White British group [RR 0.61 (95% confidence interval, 0.57–0.65) in females, 0.57 (0.52–0.62) in males]. Within the Black group, the lowest rates were within Black Africans, [e.g. for females, RR versus White British 0.35 (0.29–0.42)]; rates in the Caribbean group were closer to but still lower than the White British population [RR 0.70 (0.64–0.75)], while those for the Black Other group were similar to the White British population before standardisation for deprivation [0.94 (0.86–1.02)], but decreased with standardisation [0.78 (0.67–0.88)].

All the more specific Asian and Black ethnic groups had lower rates of admission for self-harm than the White British group in both sexes. The effect sizes for the difference increased with standardisation for deprivation for every group except Indian females. Only the White Irish, Mixed Other and Other groups had higher rates of self-harm admission than the White British group. This difference was lost for the Mixed Other group and reduced for the Other group in both sexes after standardisation for deprivation but remained for the White Irish group.

### Repeated admissions

Of the 51,745 (92%) of individuals with ethnicity data available who had at least 1 year’s follow-up following their first admission, 6,645 (12.8%) were readmitted for self-harm within a year. Rate ratios for readmission by ethnicity are shown in Table 2. When five ethnic groups were used, the Mixed group were more likely to be readmitted than the White group [adjusted RR 1.15 (1.03–1.29)], while the Asian, Black and Other groups were less likely to be readmitted. When 16 ethnic groups were used there were differences within broader groups. White Irish people were more likely to be readmitted than White British 1.43 (1.25–1.64) while White Other people were less likely to be 0.51 (0.46–0.57). Mixed White and African people were less likely to be admitted than White British, whilst all other Mixed groups were more likely to be. All the Black and Asian ethnic groups were less likely to be readmitted than White British people. The Black Other group, who were the most likely Black group to be admitted, were the least likely to be readmitted.

### Sensitivity analysis comparing ED attendances and admissions

There were 12,577 first ED attendances in the comparison area in 2009–2016, 12,289 (97.7%) had data on ethnicity. Supplementary table 4 compares rates and rate ratios by ethnicity for these attendances to those for the 4699 first admissions in the HES APC dataset for the same area and time period. All the effect sizes comparing other ethnic groups to White British were significant and in the same direction in both datasets, with lower rates in the White Other, Asian, Black and Mixed groups and higher rates in the Other group.

## Discussion

In this study, we found substantial variation in rates of general hospital admission for self-harm between different ethnic groups in London. Black and Asian people had lower rates of admission than the White population in both sexes and across all age groups. These groups were also less likely to be readmitted within a year. These differences were not explained by deprivation: standardisation for IMD had only a modest impact on rates which increased the differences in rates between the Black and Asian groups and the White group. The picture for the Mixed group was more complicated: overall rates were lower than the White group at all ages for both sexes, once standardised for deprivation. However, rates of readmission in the mixed group were higher than in the White group. There were also differences by ethnicity in the age at which self-harm peaked for men, with Black and Mixed ethnicity men having higher rates in the 25–49 age group whilst all other groups saw rates peak in people below 25.

The extent to which rates of admission following self-harm differed from those in the White British population varied considerably for more detailed ethnic groups within the broad categories. Within the White group, the Irish population had higher rates than the British population, while the White Other group had much lower rates. There were also differences between the Black groups: in both sexes Black Africans had lower rates relative to the White British group than the Black Caribbean group, who in turn had lower rates than the Black Other group. These findings highlight the importance of disaggregating data within broad ethnic categories. The Black African population is the fastest-growing minority group in the UK, doubling between 1991 and 2001, and again by 2011 [23] so that it is now the largest Black group. Likewise, the White Other group has grown rapidly in recent decades and is the largest minority group in London and the UK as a whole. However, there is little research evidence available to understand self-harm rates in either of these populations. The last community survey that reported suicidal behaviours by ethnicity in the UK, the EMPIRIC study from the late 1990s, looked specifically at the Black Caribbean and White Irish groups but had insufficient numbers to show differences in odds of self-harm [24]. More recent studies using service use data have combined the Black African group with Black Caribbean and Black Other, a heterogenous group, about half of whom identified their ethnicity as Black British in the 2011 census. Similarly, all White ethnicities tend to be combined together and used a reference group. This study suggests the experiences of these separate groups may be quite different, and that the White Other group may have more in common with other ethnic minority groups than the White British group for this outcome.

### Comparison with previous studies

Our findings of lower rates of self-harm in South Asians than White British people, for both men and women, confirms similar findings in a study using ED data from Manchester, Derby and Oxford for 2001–2006 [9], in contrast to earlier studies which found higher rates of self-harm in South Asian women [7]. Cooper et al. [9] suggested this difference may be due to different South Asian populations being included in different studies. This study found similar, lower rates of self-harm in Indian, Pakistani, Bangladeshi and Other Asian groups suggesting a pattern across all South Asian populations. However, the migration status and socio-economic position of these populations may be changing over time. It may also be that greater attention and service provision for these populations based on previously high rates has been beneficial.

This study also found lower rates of self-harm in Black women than White women at all ages, in contrast to previous studies’ findings of raised rates in young Black women [9, 10]. The authors of these studies suggested that higher rates may be due to greater socio-economic adversity affecting this group. The Black population of London in this study is disproportionately concentrated in more deprived areas so it seems unlikely that the difference could be explained by the Black women included in this study being less deprived than in previous studies. Indeed, adjusting for area socio-economic deprivation lowered rates further in comparison to White women. Another possibility lies in the differences seen between different Black groups described above. For women, the Black Other group’s rate of self-harm admission was similar to the White British group before standardisation for deprivation [RR 0.94 (0.86–1.02)]. It may be the Black group in previous studies, which did not adjust for deprivation, contained different proportions of different Black ethnicities and this study partly reflects the changing make-up of the Black population in London. An additional consideration is the role of ethnic density in protecting the mental health of individuals from ethnic minorities. Living in areas with a higher proportion of people of the same ethnicity has been found to be associated with lower rates of self-harm [25] and suicide [26] for individuals from ethnic minorities. The lower rates of self-harm in ethnic minority groups in this study may partly reflect this.

It is important to also note that the previous studies referenced used data on ED attendances while this study was restricted to admissions. It may be that the likelihood of admission following an ED presentation with self-harm varies by ethnicity. This could be either because of differential treatment of people from different ethnic groups within the same hospitals or because of differences in admission practices between hospitals which serve different populations. For example, work in South East London has found substantial differences in admission practices between hospitals, with the hospital least likely to admit following self-harm serving the most deprived and ethnically diverse areas [27]. The sensitivity analysis using ED data found lower rates of attendance following self-harm in the non-British White, Black, Asian and Mixed groups compared to the White British group, mirroring the findings from the admissions data. However, this only reflects the experience in a small part of the total study area and does not rule out a role for admission practices.

Some of our findings confirm those of previous research. The White Irish population in the UK have previously been found to experience worse health outcomes, including for psychological distress [28], which have persisted across generations, and also have higher suicide rates in men [29]. Previous studies have suggested that much of this health inequality can be explained by experiences of material deprivation in childhood [28]. In this study, the current deprivation of the area that people lived did not explain higher rates of self-harm in the Irish population, however, this is not an individual level measure, nor does it necessarily capture the conditions someone lived in growing up, hence different exposure to deprivation over the life course may still explain differences in rates.

A broader question is why there appear to be lower rates of admission for self-harm amongst most of the ethnic groups investigated when compared to the White British population, despite their greater exposure to socio-economic stressors. In their cultural model of suicide, Chu et al. [5] suggest several points at which an individual’s cultural context, particularly their ethnicity, may impact on their risk of self-harm and suicide. The stressors most associated with the risk of self-harm and suicidality may vary between groups, so the understanding of risk factors developed from suicide and self-harm research that has overwhelmingly been based on White populations may not generalise well to minority groups. Religious sanctions around suicide and self-harm may also make self-harm a less acceptable response to stressors for some groups, either making them less likely to self-harm or more likely to hide having done so. However, the pattern of lower rates in virtually all minority groups, despite their heterogeneity, should be a caution against locating explanations solely within their cultures [5]. The common experience amongst these groups is a position of a structural disadvantage as a minority within the UK [30], which could also impact the ways in which people feel able to express distress and the likelihood of their accessing help [31].

Rates of self-harm may appear lower in some ethnic groups due to differing “idioms of distress”, resulting in distress being expressed in different ways [4]. This study, like most others examining rates of self-harm by ethnicity in the UK, uses a definition of self-harm as intentional self-injury or poisoning that excludes other risk taking or harmful behaviours that could be more common responses to distress in some ethnic groups. Hospital data also relies on people presenting to services following self-harm and clinicians recognising and recording a presentation to services as self-harm. There is evidence from community surveys that people of the Black Caribbean and South Asian ethnicities were less likely to seek professional help following self-harm [24]. Studies from the USA have also found that African Americans are less likely to present clinicians with the expected “classic signs” when suicidal [4]. Research in both the UK [32] and USA [33] has suggested that suicides in ethnic minorities are less likely to be identified as such and more likely to be found of undetermined intent or misattributed to accidents, raising the possibility that similar misclassification occurs for non-fatal acts of self-harm seen in hospitals.

### Strengths and limitations

This study is strengthened by being based on a large dataset containing self-harm admissions for nearly 60,000 individuals from an underlying population of over 8 million with a very high level of completeness for the ethnicity variable at 94%. London is the most ethnically diverse region of England, with only 46% of its population identifying as White British at the 2011 census compared to 81% for England and Wales overall. There are likely to be similarities to other large urban centres in England: Birmingham and Manchester for example have similar proportions of their populations identifying as White, although there are differences, in particular, all the Black ethnic groups and White Other form a larger proportion of London’s population than other British cities [34]. The ethnic diversity within London allowed us to examine rates for more specific ethnic groups than previous studies and in doing so reveal important variations in rates within broad ethnic categories. However, the experiences of individuals from ethnic minority groups within the city cannot be assumed to be representative for the country as a whole.

An important limitation of this study is that we had to rely on data from the census in 2011 to provide denominator populations by ethnicity when calculating rates of self-harm. This was the best source of data available to provide the level of detail required to standardise rates of self-harm admission. However, the proportion of London’s population who are from ethnic minorities increased substantially between the 2001 and 2011 censuses and is likely to have continued increasing in the years since [23]. Given this, in the later years of the study period, the denominator used to calculate rates of self-harm admission for some ethnic minority groups may have been underestimated, making the rates calculated appear higher. The effect sizes for almost all ethnic minority groups, which show lower rates of self-harm admission than the White British population, may then be an underestimate of the difference.

The findings of the study are also limited by our dependence on service use data. The rates calculated only represent differences in admissions to hospital following self-harm. While admission may represent episodes of self-harm with more severe physical health consequences, they will also be affected by differences in help-seeking and admission practices between different ethnic groups. Using routine data also means that our definition of ethnicity comes from medical records. Ideally, ethnicity would be based on individuals’ self-identification. We cannot know whether the individuals in the study were always asked what they considered their ethnicity to be, for some it may have been assigned by hospital staff. The categories of ethnicity, while more specific than previous studies, are still heterogenous and may mask important variation within categories. The Other and Mixed ethnic groups (the largest subgroup of which is Mixed Other) in particular may not represent coherent groups with similar racialised experiences and are difficult to draw conclusions about from this data.

There are other individual-level variables that are likely to impact the risk of self-harm admission and readmission and may do so differently for different ethnic groups, for example, the presence of a psychiatric history or substance misuse. We were not able to adjust for these because they are not recorded in HES data. We adjusted for socio-economic position using the deprivation of individuals’ place of residence. This will not be an accurate reflection of individual deprivation for all people. An analysis using individual-level variables for socio-economic position, as well as migration status would allow greater exploration of the role of these factors in self-harm.

## Conclusions

This study demonstrates that there are substantial variations in the rates of admission for self-harm between different ethnic minority groups in the UK, and different patterns by age within ethnic groups, with rates in Black and Mixed ethnicity men peaking in the 25–44 age group, contrary to the general pattern of higher rates in those aged under 25. While the White Irish group continues to have the highest rates of self-harm, we found lower rates for all South Asian and Black ethnicities in both sexes than their White British counterparts, in contrast to the findings for women in these groups in earlier studies. People of Mixed ethnicity may be at greater risk readmission for self-harm following an initial episode than other ethnic groups. While it is hard to disentangle the effects of different levels of service use and the potential impact of misclassification using routine data, this study highlights that we should not be relying on studies and surveys from previous decades for our understanding of self-harm in minority communities. We should be using more specific ethnic categories and considering migration status in self-harm research to avoid masking differences within large, heterogenous ethnic groups.

## Supplementary Material

Supplementary Information The online version contains supplementary material available at https://doi.org/10.1007/s00127-021-02087-9

## Figures and Tables

**Table 1:** Rates of first admission for self-harm by ethnicity and sex in London 2008–2018

Ethnicity	First admissions (%)	2011 pop (1000 s)	Crude rate /10,000py	Standardised for age			Standardised for age and IMD	
				Standardised rate /10,000py	Standardised RR*		Standardised rate /10,000py	Standardised RR*
Females								
White								
British	18,777 (53.5)	1633	11.5 (11.3–11.7)	12.5 (12.3–12.7)	1.00 (ref)		13.9 (13.7–14.1)	1.00 (ref)
Irish	855 (2.4)	87	9.8 (9.1–10.5)	13.4 (12.4–14.5)	1.07 (0.99–1.15)		15.8 (14.6–17.2)	1.14 (1.06–1.22)
Other	3276 (9.3)	491	6.7 (6.5–6.9)	7.6 (7.3–7.9)	0.61 (0.57–0.65)		7.3 (7.0–7.6)	0.52 (0.49–0.56)
Mixed								
White and	709 (2.0)	43	16.6 (15.4–17.8)	12.4 (11.3–13.5)	0.99 (0.90–1.08)		11.2 (10.2–12.3)	0.80 (0.71–0.90)
Caribbean								
White and	213 (0.6)	22	9.6 (8.4–11.0)	7.9 (6.7–9.3)	0.63 (0.47–0.79)		6.6 (5.6–7.9)	0.48 (0.31–0.65)
African								
White and	186 (0.5)	33	5.7 (4.9–6.6)	4.5 (3.8–5.3)	0.36 (0.20–0.53)		4.1 (3.4–4.9)	0.30 (0.12–0.48)
Asian								
Other	929 (2.6)	43	21.4 (20.0–22.8)	16.6 (15.4–17.8)	1.32 (1.25–1.40)		15.0 (13.9–16.2)	1.08 (1.00–1.16)
Asian								
Indian	1544 (4.4)	236	6.5 (6.2–6.9)	6.6 (6.3–6.9)	0.53 (0.48–0.58)		7.5 (7.1–8.0)	0.54 (0.48–0.60)
Pakistani	760 (2.2)	83	9.2 (8.5–9.9)	7.8 (7.2–8.4)	0.62 (0.55–0.70)		7.2 (6.7–7.8)	0.52 (0.44–0.60)
Bangladeshi	707 (2.0)	82	8.6 (8.0–9.3)	6.0 (5.5–6.5)	0.48 (0.40–0.56)		6.1 (5.2–7.1)	0.44 (0.29–0.59)
Other	1413 (4.0)	173	8.2 (7.8–8.6)	7.8 (7.4–8.2)	0.62 (0.57–0.68)		7.6 (7.2–8.0)	0.55 (0.49–0.60)
Black								
Caribbean	1413 (4.0)	171	8.3 (7.8–8.7)	8.7 (8.2–9.2)	0.70 (0.64–0.75)		8.9 (8.3–9.5)	0.64 (0.57–0.70)
African	1259 (3.6)	241	5.2 (4.9–5.5)	4.4 (4.2–4.7)	0.35 (0.29–0.42)		4.2 (3.8–4.6)	0.30 (0.21–0.39)
Other	869 (2.5)	61	14.2 (13.2–15.1)	11.8 (10.9–12.7)	0.94 (0.86–1.02)		10.8 (9.7–12.0)	0.78 (0.67–0.88)
Other								
Chinese	156 (0.4)	62	2.5 (2.1–2.9)	2.6 (2.2–3.1)	0.21 (0.03–0.39)		2.8 (2.3–3.3)	0.20 (0.03–0.36)
Any other	2056 (5.9)	109	18.9 (18.1–19.7)	16.6 (15.9–17.4)	1.33 (1.28–1.38)		17.0 (16.2–17.8)	1.22 (1.17–1.27)
Male								
White								
British	12,379 (59.0)	1620	7.6 (7.5–7.8)	8.0 (7.8–8.1)	1.00 (ref)		9.0 (8.8–9.2)	1.00 (ref)
Irish	706 (3.4)	81	8.7 (8.0–9.3)	9.1 (8.4–9.9)	1.14 (1.06–1.23)		10.3 (9.4–11.3)	1.15 (1.06–1.24)
Other	1942 (9.2)	438	4.4 (4.2–4.6)	4.5 (4.3–4.8)	0.57 (0.52–0.62)		4.4 (4.2–4.7)	0.49 (0.44–0.55)
Mixed								
White and	223 (1.1)	38	5.8 (5.1–6.6)	6.1 (5.2–7.1)	0.76 (0.61–0.91)		5.7 (4.8–6.7)	0.63 (0.47–0.79)
Caribbean								
White and	77 (0.4)	20	3.8 (3.0–4.8)	4.3 (3.2–5.7)	0.54 (0.26–0.82)		3.9 (2.9–5.3)	0.44 (0.14–0.73)
African								
White and	85 (0.4)	34	2.5 (2.0–3.1)	2.6 (2.0–3.3)	0.33 (0.08–0.57)		2.5 (1.9–3.2)	0.28 (0.03–0.53)
Asian								
Other	358 (1.7)	39	9.3 (8.3–10.3)	9.0 (8.0–10.1)	1.12 (1.01–1.24)		7.9 (7.0–8.9)	0.88 (0.76–1.00)
Asian								
Indian	895 (4.3)	244	3.7 (3.4–3.9)	3.7 (3.4–3.9)	0.46 (0.39–0.53)		4.0 (3.7–4.3)	0.44 (0.37–0.52)
Pakistani	297 (1.4)	97	3.1 (2.7–3.4)	2.8 (2.4–3.1)	0.35 (0.23–0.46)		2.8 (2.4–3.1)	0.31 (0.18–0.44)
Bangladeshi	219 (1.0)	88	2.5 (2.2–2.8)	2.1 (1.8–2.5)	0.27 (0.11–0.42)		2.0 (1.6–2.5)	0.23 (0.02–0.44)
Other	874 (4.2)	163	5.4 (5.0–5.7)	5.2 (4.8–5.5)	0.65 (0.57–0.72)		5.1 (4.7–5.4)	0.57 (0.49–0.64)
Black								
Caribbean	586 (2.8)	130	4.5 (4.2–4.9)	5.1 (4.6–5.5)	0.63 (0.55–0.72)		4.6 (4.1–5.0)	0.51 (0.41–0.60)
African	532 (2.5)	207	2.6 (2.4–2.8)	2.5 (2.3–2.8)	0.32 (0.22–0.41)		2.4 (2.1–2.7)	0.27 (0.15–0.39)
Other	407 (1.9)	60	6.8 (6.2–7.5)	6.9 (6.1–7.6)	0.86 (0.75–0.97)		6.2 (5.4–7.1)	0.69 (0.56–0.82)
Other								
Chinese	54 (0.3)	52	1.0 (0.8–1.3)	1.0 (0.8–1.4)	0.13 (0.01–0.41)		1.1 (0.8–1.4)	0.12 (0.01–0.39)
Any other	1361 (6.5)	126	10.8 (10.3–11.4)	10.4 (9.9–11.0)	1.31 (1.25–1.37)		10.3 (9.7–10.9)	1.14 (1.08–1.20)

*RR* Rate ratio, *IMD* Index of Multiple Deprivation *p< 0.001

**Table 2:** Readmission for self-harm in the 12 months following first admission for self-harm, by ethnicity in London 2008–2018

Ethnicity	First admissions	Readmitted (%)	Univariate RR* (95% CI)	Adjusted^a^ RR* (95% CI)
*White*	35,109	4983 (14.2)	1.00 (ref)	1.00 (ref)
British	28,899	4281 (14.8)	1.00 (ref)	1.00 (ref)
Irish	1486	281 (18.9)	1.39 (1.21–1.59)	1.43 (1.25–1.64)
Other	4724	421 (8.9)	0.53 (0.48–0.59)	0.51 (0.46–0.57)
*Mixed*	2540	416 (16.4)	1.20 (1.08–1.35)	1.15 (1.03–1.29)
White and Caribbean	864	147 (17.0)	1.20 (1.00–1.44)	1.12 (0.93–1.35)
White and African	270	30 (11.1)	0.70 (0.48–1.03)	0.67 (0.46–0.99)
White and Asian	240	41 (17.1)	1.20 (0.85–1.69)	1.16 (0.82–1.63)
Other	1166	198 (17.0)	1.19 (1.02–1.40)	1.12 (0.96–1.32)
*Asian*	6218	635 (10.2)	0.66 (0.61–0.72)	0.64 (0.59–0.70)
Indian	2285	244 (10.7)	0.66 (0.57–0.76)	0.65 (0.57–0.75)
Pakistani	998	98 (9.8)	0.60 (0.48–0.74)	0.57 (0.46–0.70)
Bangladeshi	837	102 (12.2)	0.77 (0.63–0.96)	0.71 (0.58–0.88)
Other	2098	191 (9.1)	0.55 (0.47–0.64)	0.53 (0.45–0.61)
*Black*	4620	427 (9.2)	0.59 (0.53–0.65)	0.57 (0.51–0.63)
Caribbean	1833	181 (9.9)	0.60 (0.51–0.70)	0.58 (0.49–0.68)
African	1651	166 (10.1)	0.61 (0.52–0.72)	0.58 (0.49–0.69)
Other	1136	80 (7.0)	0.41 (0.32–0.51)	0.39 (0.31–0.49)
*Other*	3258	184 (5.6)	0.34 (0.29–0.39)	0.33 (0.28–0.38)
Chinese	185	15 (8.1)	0.47 (0.28–0.81)	0.45 (0.27–0.77)
Any other	3073	169 (5.5)	0.31 (0.26–0.36)	0.30 (0.25–0.35)

*CI* confidence interval, *RR* Rate ratio

* Likelihood ratio test for association ethnicity and readmission *p* < 0.0001 (both 5 and 16 groups)

a adjusted for age, sex, type of self-harm and area deprivation Broad, five-category ethnic groups shown in italics; more specific, sixteen-category ethnic groups shown in regular

## Data Availability

CP had access to all the data used in this analysis, as the named applicant on the Data Sharing Agreement with NHS Digital. This Data Sharing Agreement does not allow further sharing of this data outside of the immediate research team, however, Hospital Episode Statistics data, including all the data used in this study, is available by application to NHS Digital.
