# Supplementary material for: Variation in rates of self-harm hospital admission and re-admission by ethnicity in London: a population cohort study
Source: Soc Psychiatry Psychiatr Epidemiol. Author manuscript; Available in PMC 2021 Nov 1. (PMC8519852; doi:10.1007/s00127-021-02087-9)
Supplement: Supplementary Information [file EMS135998-supplement-Supplementary_Information.pdf]

Supplementary table 1. Population of London aged 11 years and over at the 2011 Census by ethnicity and deprivation of area of residence

Ethnicity	Total Population in 1000s, (%)	National quintile Index of Multiple Deprivation 2015 Population in 1000s (Column %)				
		Most deprived	2	3	4	Least deprived
White	***4295 (61.7)***	***758 (49.2)***	***1181 (55.7)***	***937 (63.7)***	***833 (75.0)***	***586 (82.0)***
British	3207 (46.1)	523 (33.9)	834 (39.3)	691 (47.0)	657 (59.2)	503 (70.3)
Irish	166 (2.4)	33 (2.1)	51 (2.4)	38 (2.6)	27 (2.4)	17 (2.4)
Other	921 (13.2)	202 (13.1)	297 (14.0)	208 (14.2)	148 (13.3)	66 (9.2)
Mixed	***294 (4.2)***	***83 (5.4)***	***97 (4.6)***	***59 (4.0)***	***36 (3.2)***	***19 (2.7)***
White & Caribbean	84 (1.2)	28 (1.8)	29 (1.4)	15 (1.0)	8 (0.7)	4 (0.6)
White & African	47 (0.7)	17 (1.1)	16 (0.8)	8 (0.5)	4 (0.4)	2 (0.3)
White & Asian	74 (1.1)	14 (0.9)	22 (1.0)	17 (1.2)	13 (1.2)	8 (1.1)
Other	89 (1.3)	24 (1.6)	30 (1.4)	18 (1.2)	11 (1.0)	6 (0.8)
Asian	***1155 (16.6)***	***244 (15.8)***	***417 (19.7)***	***270 (18.4)***	***150 (13.5)***	***73 (10.2)***
Indian	471 (6.8)	55 (3.6)	167 (7.9)	132 (9.0)	78 (7.0)	39 (5.4)
Pakistani	179 (2.6)	35 (2.3)	79 (3.7)	40 (2.7)	17 (1.5)	8 (1.1)
Bangladeshi	171 (2.5)	88 (5.7)	56 (2.6)	17 (1.2)	7 (0.6)	3 (0.4)
Other	333 (4.8)	66 (4.3)	115 (5.4)	81 (5.5)	48 (4.3)	23 (3.2)
Black	***878 (12.6)***	***366 (23.7)***	***317 (14.9)***	***132 (9.0)***	***47 (4.2)***	***17 (2.4)***
Caribbean	294 (4.2)	110 (7.1)	109 (5.1)	51 (3.5)	18 (1.6)	7 (1.0)
African	456 (6.6)	202 (13.1)	161 (7.6)	62 (4.2)	22 (2.0)	8 (1.1)
Other	128 (1.8)	54 (3.5)	46 (2.2)	19 (1.3)	7 (0.6)	2 (0.3)
Other	***338 (4.9)***	***91 (5.9)***	***110 (5.2)***	***72 (4.9)***	***45 (4.1)***	***20 (2.8)***
Chinese	106 (1.5)	22 (1.4)	32 (1.5)	25 (1.7)	17 (1.5)	9 (1.3)
Any other	232 (3.3)	69 (4.5)	78 (3.7)	47 (3.2)	28 (2.5)	11 (1.5)

Supplementary table 2. International classification of diseases revision 10 codes for intentional self-harm.

Code	Definition
**Poisoning**	
X60	Intentional self-poisoning by and exposure to nonopioid analgesics, antipyretics and antirheumatics
X61	Intentional self-poisoning by and exposure to antiepileptic, sedative-hypnotic,
X62	Intentional self-poisoning by and exposure to narcotics and psychodysleptics [hallucinogens], not elsewhere classified
X63	Intentional self-poisoning by and exposure to other drugs acting on the autonomic
X64	Intentional self-poisoning by and exposure to other and unspecified drugs,
X65	Intentional self-poisoning by and exposure to alcohol
X66	Intentional self-poisoning by and exposure to organic solvents and halogenated hydrocarbons and their vapours
X67	Intentional self-poisoning by and exposure to other gases and vapours
X68	Intentional self-poisoning by and exposure to pesticides
X69	Intentional self-poisoning by and exposure to other and unspecified chemicals and
**Injury**	
X72	Intentional self-harm by handgun discharge
X73	Intentional self-harm by rifle, shotgun and larger firearm discharge
X74	Intentional self-harm by other and unspecified firearm discharge
X75	Intentional self-harm by explosive material
X76	Intentional self-harm by smoke, fire and flames
X77	Intentional self-harm by steam, hot vapours and hot objects
X78	Intentional self-harm by sharp object
X79	Intentional self-harm by blunt object
**Other**	
X70	Intentional self-harm by hanging, strangulation and suffocation
X71	Intentional self-harm by drowning and submersion
X80	Intentional self-harm by jumping from a high place
X81	Intentional self-harm by jumping or lying before moving object
X82	Intentional self-harm by crashing of motor vehicle
X83	Intentional self-harm by other specified means
**Unspecified**	
X84	Intentional self-harm by unspecified means

Source: International Statistical Classification of Diseases and Related Health Problems 10th Revision, 2016 version. World Health Organisation, Geneva.

Supplementary table 3. Rates of first admission for self-harm by ethnicity, age and sex in London 2008-2018

Ethnicity	First admissions (n=56,117)	Person years (1000s)	Rate /10,000py (95% CI)	Rate ratio (95% CI)	Standardised for IMD	
					Standardised rate /10,000py (95% CI)	Standardised RR (95% CI)
White						
**Females**	*22908*	*21777*	*11.2 (11.0-11.3)*	*1.00 (ref)*	*12.0 (11.9-12.2)*$^{+}$	*1.00 (ref)*
11-24	8842	3443	25.7 (25.2-26.2)	1.00 (ref)	26.3 (25.8-26.9)	1.00 (ref)
25-49	9777	10268	9.5 (9.3-9.7)	1.00 (ref)	9.9 (9.7-10.1)	1.00 (ref)
50-64	2811	4043	7.0 (6.7-7.2)	1.00 (ref)	7.7 (7.4-8.0)	1.00 (ref)
65+	1478	4023	3.7 (3.5-3.9)	1.00 (ref)	3.8 (3.6-4.0)	1.00 (ref)
**Males**	*15027*	*21169*	*7.2 (7.0-7.3)*	*1.00 (ref)*	*7.8 (7.6-7.9)*$^{+}$	*1.00 (ref)*
11-24	3121	3476	9.0 (8.7-9.3)	1.00 (ref)	9.3 (9.0-9.7)	1.00 (ref)
25-49	8218	10611	7.7 (7.6-7.9)	1.00 (ref)	8.2 (8.0-8.3)	1.00 (ref)
50-64	2510	4043	6.2 (6.0-6.5)	1.00 (ref)	7.0 (6.7-7.2)	1.00 (ref)
65+	1178	3039	3.9 (3.7-4.1)	1.00 (ref)	4.2 (4.0-4.5)	1.00 (ref)
Mixed						
**Females**	*2037*	*1510*	*10.9 (10.4-11.5)*	*0.98 (0.93-1.03)*$^{+}$	*9.9 (9.4-10.4)*$^{+}$	*0.82 (0.77-0.88)*
11-24	1273	651	19.6 (18.5-20.7)	0.76 (0.72-0.81)	18.8 (17.8-19.9)	0.72 (0.66-0.78)
25-49	665	673	9.9 (9.1-10.7)	1.04 (0.96-1.12)	9.2 (8.5-9.9)	0.92 (0.84-1.00)
50-64	79	122	6.5 (5.1-8.1)	0.93 (0.74-1.16)	6.3 (5.0-7.9)	0.83 (0.60-1.05)
65+	20	64	3.1 (1.9-4.8)	0.86 (0.53-1.29)	3.2 (2.0-5.0)	0.85 (0.40-1.29)
**Males**	*743*	*1425*	*5.6 (5.2-6.1)*	*0.78 (0.70-0.87)*$^{+}$	*5.1 (4.7-5.6)*$^{+}$	*0.66 (0.57-0.75)*
11-24	268	653	4.1 (3.6-4.6)	0.46 (0.40-0.52)	4.0 (3.5-4.5)	0.43 (0.30-0.56)
25-49	409	618	6.6 (6.0-7.3)	0.85 (0.77-0.94)	6.2 (5.6-6.9)	0.76 (0.66-0.86)
50-64	52	100	5.2 (3.9-6.8)	0.84 (0.63-1.09)	4.9 (3.6-6.4)	0.70 (0.42-0.98)
65+	14	54	2.6 (1.4-4.3)	0.67 (0.38-1.09)	2.7 (1.4-4.5)	0.63 (0.10-1.17)
Asian						
**Females**	*4424*	*5709*	*7.1 (6.9-7.3)*	*0.63 (0.60-0.67)*$^{+}$	*7.0 (6.8-7.2)*$^{+}$	*0.58 (0.55-0.62)*
11-24	2155	1297	16.6 (15.9-17.3)	0.65 (0.62-0.68)	16.1 (15.4-16.8)	0.61 (0.56-0.66)
25-49	1954	3003	6.5 (6.2-6.8)	0.68 (0.65-0.72)	6.3 (6.0-6.6)	0.64 (0.59-0.69)
50-64	245	913	2.7 (2.4-3.0)	0.39 (0.34-0.44)	2.7 (2.3-3.0)	0.35 (0.22-0.48)
65+	70	496	1.4 (1.1-1.8)	0.38 (0.30-0.49)	1.3 (1.0-1.7)	0.35 (0.11-0.60)
**Males**	*2285*	*5836*	*3.7 (3.6-3.9)*	*0.52 (0.47-0.56)*$^{+}$	*3.7 (3.5-3.8)*$^{+}$	*0.47 (0.43-0.52)*
11-24	632	1417	4.5 (4.1-4.8)	0.50 (0.46-0.54)	4.3 (4.0-4.7)	0.46 (0.37-0.55)
25-49	1326	3124	4.2 (4.0-4.5)	0.55 (0.52-0.58)	4.1 (3.8-4.3)	0.50 (0.44-0.56)
50-64	233	836	2.8 (2.4-3.2)	0.45 (0.39-0.51)	2.8 (2.4-3.2)	0.40 (0.26-0.54)
65+	94	459	2.0 (1.7-2.5)	0.53 (0.43-0.65)	2.0 (1.6-2.5)	0.48 (0.26-0.69)
Black						
**Females**	*3541*	*4751*	*6.8 (6.6-7.0)*	*0.61 (0.57-0.64)*$^{+}$	*6.5 (6.3-6.8)*$^{+}$	*0.54 (0.50-0.59)*
11-24	2004	1233	16.2 (15.5-17.0)	0.63 (0.60-0.66)	16.8 (15.8-17.9)	0.64 (0.57-0.70)
25-49	1303	2446	5.3 (5.0-5.6)	0.56 (0.53-0.59)	5.2 (4.8-5.6)	0.52 (0.45-0.59)
50-64	185	680	2.7 (2.3-3.1)	0.39 (0.34-0.45)	2.5 (2.1-2.9)	0.32 (0.14-0.50)
65+	49	392	1.2 (0.9-1.7)	0.34 (0.25-0.45)	1.8 (1.2-2.5)	0.46 (0.08-0.84)
**Males**	*1525*	*4027*	*3.9 (3.7-4.1)*	*0.55 (0.50-0.60)*$^{+}$	*3.7 (3.4-3.9)*$^{+}$	*0.47 (0.41-0.54)*
11-24	426	1226	3.5 (3.2-3.8)	0.39 (0.35-0.43)	3.8 (3.3-4.4)	0.41 (0.27-0.55)
25-49	886	1964	4.5 (4.2-4.8)	0.58 (0.54-0.62)	4.3 (4.0-4.7)	0.53 (0.44-0.62)
50-64	164	534	3.1 (2.6-3.6)	0.50 (0.42-0.58)	3.0 (2.5-3.6)	0.43 (0.24-0.63)
65+	49	303	1.6 (1.2-2.1)	0.42 (0.31-0.55)	1.7 (1.2-2.3)	0.40 (0.05-0.74)
Other						
**Females**	*2212*	*1656*	*11.7 (11.2-12.2)*	*1.05 (1.00-1.09)*$^{+}$	*12.1 (11.6-12.7)*$^{+}$	*1.01 (0.96-1.05)*
11-24	1151	381	30.2 (28.5-32.0)	1.18 (1.11-1.25)	30.0 (28.2-31.8)	1.14 (1.07-1.20)
25-49	888	915	9.7 (9.1-10.4)	1.02 (0.95-1.09)	9.6 (9.0-10.3)	0.96 (0.89-1.03)
50-64	131	248	5.3 (4.4-6.3)	0.76 (0.64-0.90)	5.3 (4.5-6.3)	0.70 (0.52-0.87)
65+	42	112	3.8 (2.7-5.1)	1.03 (0.74-1.37)	3.8 (2.8-5.2)	1.00 (0.70-1.31)
**Males**	*1415*	*1727*	*7.6 (7.2-8.0)*	*1.06 (1.00-1.11)*$^{+}$	*7.6 (7.2-8.0)*$^{+}$	*0.98 (0.92-1.04)*
11-24	407	401	10.2 (9.2-11.2)	1.13 (1.02-1.25)	10.0 (9.0-11.0)	1.07 (0.96-1.17)
25-49	834	983	8.5 (7.9-9.1)	1.10 (1.02-1.18)	8.1 (7.6-8.7)	1.00 (0.92-1.07)
50-64	129	239	5.4 (4.5-6.4)	0.87 (0.73-1.03)	5.2 (4.4-6.2)	0.75 (0.57-0.93)
65+	45	104	4.3 (3.2-5.8)	1.12 (0.82-1.49)	4.3 (3.2-5.8)	1.03 (0.73-1.33)

Supplementary Table 4. First admission and first Emergency Department attendance following self-harm by ethnicity in Lambeth, Southwark, Lewisham and Croydon boroughs, London 2009-2016

	First ED attendances 2009-2016					First admissions 2009-2016				
**Ethnicity**	**n (%)**	**py (1000s)**	**Crude rate /10,000py**	**Rate ratio (95% CI)**	**Standardised RR (95% CI)**	**n (%)**	**py (1000s)**	**Crude rate /10,000py**	**Rate ratio (95% CI)**	**Standardised RR (95% CI)**
White British	6610 (53.8)	2683	24.6 (24.0-25.2)	1.00 (ref)	1.00 (ref)	2647 (56.3)	2683	9.9 (9.5-10.2)	1.00 (ref)	1.00 (ref)
White Non-British	1268 (10.3)	846	15.0 (14.2-15.8)	0.61 (0.57-0.65)	0.57 (0.54-0.61)	462 (9.8)	846	5.5 (5.0-6.0)	0.55 (0.50-0.61)	0.52 (0.47-0.57)
Mixed	507 (4.1)	366	13.9 (12.7-15.1)	0.56 (0.51-0.62)	0.39 (0.36-0.43)	219 (4.7)	366	6.0 (5.2-6.8)	0.61 (0.53-0.70)	0.42 (0.37-0.48)
Asian	655 (5.3)	673	9.7 (9.0-10.5)	0.39 (0.36-0.43)	0.34 (0.31-0.37)	281 (6.0)	673	4.2 (3.7-4.7)	0.42 (0.37-0.48)	0.36 (0.32-0.41)
Black	2292 (18.7)	1499	15.3 (14.7-15.9)	0.62 (0.59-0.65)	0.51 (0.49-0.54)	773 (16.5)	1499	5.2 (4.8-5.5)	0.52 (0.48-0.57)	0.43 (0.40-0.47)
Other	957 (7.8)	156	61.3 (57.6-65.3)	2.49 (2.33-2.66)	2.12 (1.98-2.27)	317 (6.7)	156	20.3 (18.2-22.7)	2.06 (1.83-2.31)	1.76 (1.56-1.97)

RR - rate ratio, CI - confidence interval, py- person years
